# DETERMINANTS OF POSITIVE MARITAL QUALITY AMONG OLDER HISPANIC ADULTS IN THE US

**DOI:** 10.1093/geroni/igad104.3062

**Published:** 2023-12-21

**Authors:** Jaminette Nazario, Takashi Yamashita, Jennifer Bulanda, J Scott Brown

**Affiliations:** University of Maryland, Baltimore County, Baltimore, Maryland, United States; University of Maryland, Baltimore County, Baltimore, Maryland, United States; Miami University, Oxford, Ohio, United States; Miami University, Oxford, Ohio, United States

## Abstract

Marital Quality can significantly influence a variety of health and well-being outcomes in later life. Yet, relatively little is known about how marital quality is determined across subpopulations given their cultural backgrounds. Social networks and familial relationships are culturally important to Hispanic adults over their life course. Also, the unique characteristics (e.g., high % of immigrants) of older Hispanic adults in the U.S. may make their determinants of marital quality different from others. Considering approximately one in three older Hispanic adults is an immigrant in the U.S., this subpopulation may have relatively small social networks as their family members live in their countries of origin. As such, sub-groups of married older Hispanic adults may depend more on their marital relationships for social and cultural support, compared to other racial and ethnic groups. Framed by socioemotional selectivity theory, this study aimed to investigate determinants of marital quality among older Hispanic adults (age 51 and older). Nationally representative samples of older Hispanic adults (n = 694) were derived from the 2010 Health and Retirement Study (HRS). Results from linear regression analysis indicated that women (b = -0.16, p < 0.05) reported lower positive marital quality than men. There was also a statistically significant negative association between depressive symptoms (b = -0.09, p < 0.05) and positive marital quality. This study further evaluated other marital quality determinants, gender differences, and possible sociocultural explanations.

